# Photoinitiators for Medical Applications—The Latest Advances

**DOI:** 10.3390/molecules29163898

**Published:** 2024-08-17

**Authors:** Monika Dzwonkowska-Zarzycka, Alina Sionkowska

**Affiliations:** 1 Department of Organic Chemistry, Faculty of Chemical Technology and Engineering, Bydgoszcz University of Science and Technology, Seminaryjna 3, 85-326 Bydgoszcz, Poland; 2 Department of Biomaterials and Cosmetic Chemistry, Faculty of Chemistry, Nicolaus Copernicus University in Torun, Gagarina 7 Street, 87-100 Torun, Poland

**Keywords:** photoinitiator, medical application, light, photopolymerization

## Abstract

Photopolymerization is becoming increasingly popular in industry due to its copious advantages. The vital factor in the entire pre-polymerization formulation is the presence of photoinitiators. Depending on the application, photoinitiators have different features. Hence, scientists are particularly interested in developing new photoinitiators that can expand the scope of applications and be used to create products with the features demanded by current trends. This brief review summarizes the photoinitiators used in dental materials and hydrogels and those obtained from natural and synthetic sources.

## 1. Introduction

The importance of light does not need to be explained too much. Most of us know its role and significance. Photosynthesis is one of the first essential-for-life processes we learn of during our education. Thanks to this, as a result of a series of reactions, some organisms (such as plants or cyanobacteria) are able to produce from inorganic substances (CO_2_, H_2_O) an organic substance (C_6_H_12_O_6_—glucose) that is the basic source of energy for life and O_2_—oxygen, which is also important for our existence [1]. Access to light shapes our daily rhythm: light helps us know when to rest/sleep, wake up, and work/study. Our bodies have also adapted to produce hormones that are responsible for our functioning in its absence (melatonin) or presence (serotonin). Light deficiency or low exposure can cause depression [2]. Of course, the energy sources currently used (coal, crude oil) primarily consist of accumulated light energy. Nowadays, we increasingly use light for everyday processes (photolithography, printing, photos) [3]. Therefore, in addition to the fact that the use of light is ecologically and economically justified, it should not be surprising that there is such a growing interest in processes or using light as an energy source in every field of industry.

Accordingly, in 1912, Ciamician put forward the thesis that basing modern chemistry on this energy source could revolutionize our economy and ecology. Furthermore, in polymer chemistry, a process that uses light can be found [4]. It is photopolymerization, which involves converting a liquid polymerization mixture into a solid under the influence of light absorption. Due to its advantages, this process is readily used in many areas of industry. These advantages include that photopolymerization can be carried out at room temperature (the reaction is initiated by breaking the energy barrier through photoexcitation). The absorption of a quantum of light by chromophores initiates electrons’ transition to a higher energy state. Relaxation can take place in the following forms: thermally, via luminescence, through changes in structure, or via the transfer of an electron to initiate another photochemical reaction. This feature is more significant for biomedical applications, where high temperatures can change or damage biological molecules (for example, peptides) and temporal control (unlike reactions initiated thermally or chemically, photopolymerization can be time-controlled). It is characterized by a quick ability to both start and stop a reaction, spatial control (unlike thermally activated reactions, where the entire heated sample is activated), and rate control (which can be achieved by manipulating light’s intensity and wavelength). In the case of photopolymerization, only molecules with chromophores that receive radiation of a specific wavelength are activated. Mainly due to this feature, it is possible to use photopolymerization in 3D technology. In contrast to chemical or thermal processes, photopolymerization is not dependent on heat or mass transfer, is solvent-free (thanks to the use of photoinitiators with good solubility in mixtures of monomers), and does not release volatile organic compounds. All these features allow for the general statement that polymerization is ecologically friendly [4,5,6].

A listing of the features of photopolymerization in comparison to thermal polymerization can be found in a 2019 publication, as presented in Table 1 [7].

To carry out the photopolymerization process, it is necessary to properly design and prepare the reaction mixture. A typical mixture consists of the following:

- An oligomer or resin (unsaturated or acrylate), which is responsible for the properties of the final polymer, consisting of 1 to 12 unit monomers;
- Monomers, which are used to increase the viscosity and flexibility of the polymer;
- Additives—for example, talc or pigments—which are responsible for lowering prices or giving colour;
- A photoinitiator, whose role is to react at a specific wavelength and form cations, anions, or radicals (depending on the types of particles formed, for which cationic or radical photopolymerization can be distinguished [8,9,10,11,12]) and then initiate the polymerization process. The mechanism of this reaction is based on the absorption of light of a specific wavelength, followed by the excitation of ground-state electrons to the excited singlet state and then the triplet state, from which they undergo decay and form active centers [13]. Photoinitiators play a crucial role in photopolymerization, hence the great interest in the design of new photoinitiator systems. It is also crucial to determine the appropriate concentration of a photoinitiator because it is the most expensive element of the photocurable mixture, and this has economic justification [14]. The requirements for photoinitiators during their design and subsequent use are a low production cost, relatively uncomplicated synthesis, good solubility in the ingredients of the reaction mixture, absorption in the visible spectrum, and high polymerization efficiency. Additionally, when used in biomedical applications, photoinitiators must have good solubility in water and no negative effects on cells [15,16]. In free radical photopolymerization, the following types of photoinitiators (PIs) can be distinguished:
  - Type I, where the dissociation of the molecule and the formation of active centers takes place through direct absorption of a quantum of light by the molecule, which is a one-component system;
  - Type II, where active centers are produced after multi-component reactions, involving the transfer of electrons or hydrogen between the co-initiator and photoinitiators after the absorption quantum of light, constituting two- or multi-component systems [8].

Juxtaposing the implementation of radical and cationic polymerization, one can notice more frequent use of free radical polymerization in the biomedical area. This is due to the disadvantages of cationic polymerization, which include the following: (1) the sensitivity of this system to humidity and the presence of water, and (2) the generation of a protonic environment, which negatively affects cells [15]. When comparing individual types of photoinitiators, it is difficult to determine which of them is dedicated more to biomedical purposes. Each has advantages and disadvantages and should be considered depending on the specific application. Considering dentistry, for instance, the advantages of type 1 are that they absorb in the shorter wavelength range, are less pigmented and improve the colour of dental resin, have high molar extinction coefficients, and improve hardness. In type 2, in turn, photoinitiators are characterized by slower photoinitiation caused by a two-step reaction. They require the presence of a co-initiator, which may have toxic properties for cells, but they often absorb in the visible range [17].

**Figure 1:**
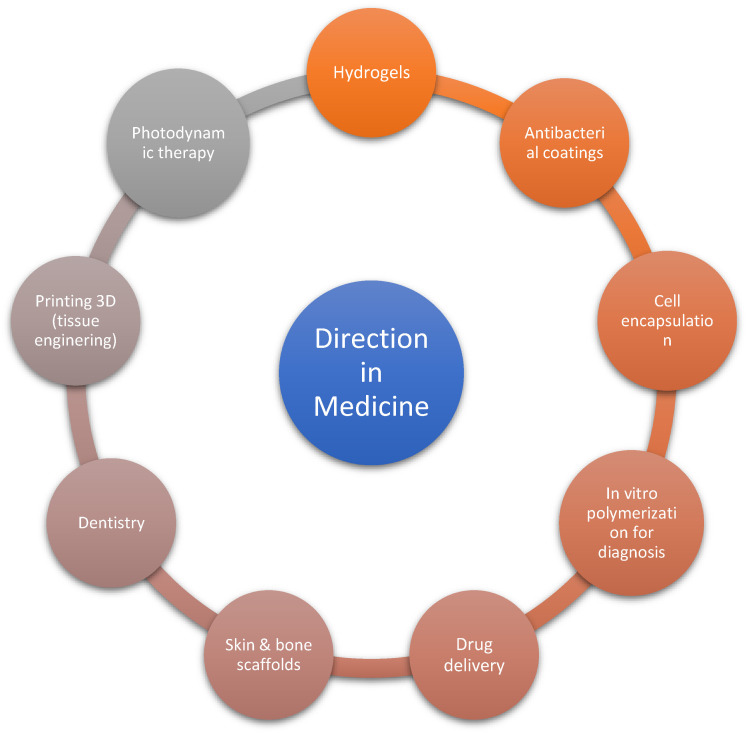
The key routes of use of photopolymerization in medicine [19,20].

The main directions of use of photopolymerization are coatings, 3D printing, adhesives, electronics, optics, pharmacy and medicine [18]. In medicine, the use of photopolymerization and photoinitiators is focused on refining several elements. These elements are presented in Figure 1.

This review article aims to attempt to summarize the photoinitiators currently in use. It focuses on those that have particular applications in medicine. General information on these photoinitiators of natural and synthetic origin was collected, thus displaying the latest research directions in this field. Nonetheless, the history of some applications is briefly reviewed as well.

## 2. Classification of Photoinitiators

### 2.1. Photoinitiators of Natural Origin

**Figure 2:**
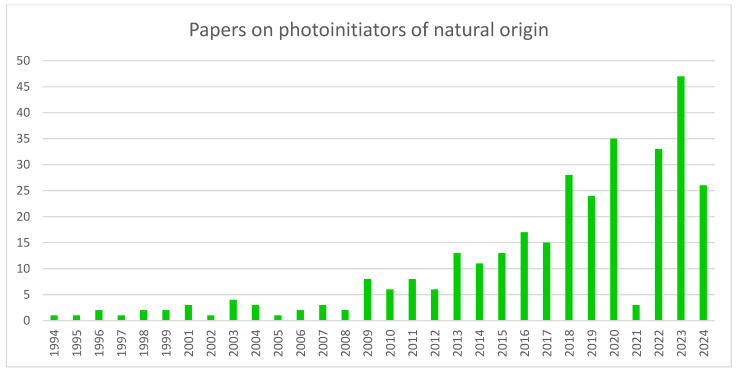
A chart showing the growing trend of interest in photoinitiators of natural origin. Made based on data from Scopus.

Substances of natural origin are a particular group of photoinitiators that is gaining more attention. Figure 2 displays the trend of work on this type of compound. As the chart below shows, the general trend over the years has been upward. The specific features of this type of photoinitiators explain this interest. The chief disadvantages of synthetic PI include the high cost of synthesis, cytotoxicity and solubility problems. The use of natural PI can reduce these problems [20].

The principal advantages of naturally derived PIs include lesser toxicity and higher biocompatibility [21]. The list of the most popular and most common photoinitiators is presented in Table 2 (below). Photoinitiators of natural origin show potential for further study and use in medical applications. The main directions of work with them should focus on increasing the penetration level of the skin–light barrier and optimization of concentration and other conditions of the photopolymerization reaction depending on the application [22].

### 2.2. Photoinitiators of Synthetic Origin

Although naturally derived photoinitiators have become increasingly significant, photoinitiators of synthetic origin cannot be overlooked. Well-known photolysis mechanisms, the ability to initiate polymerization, and developed synthesis methods provide the opportunity to use them further and improve the properties of photocurable compositions. Table 3 lists the most common photoinitiators of synthetic origin, detailing their characteristics.

The TPO (MAPO) and BAPO are the most famous photoinitiators. Their use is not limited to one field. They can be used in photopolymerization to produce coatings or inks or implemented in the biomedical industry [23]. However, the use of TPO in the biomedical field has become increasingly controversial due to ECHA’s findings from 2019, which identified TPO as a toxic photoinitiator. The outcome was a recommendation to restrict the substance application in medicine; presumably, it may be entirely banned [24]. For this reason, the germanium-based photoinitiators were proposed. In addition to the lack of toxicity, the following seem particularly worth mentioning:

- Due to the low content of germanium in the earth’s crust, the widespread use of germanium, and therefore, germanium-based photoinitiators, may be limited due to their high price and restricted to critical areas such as medicine [25],
- They are type I photoinitiators, photodissociate at the Ge-C bond [26],
- Absorption capacity is up to 480 nm [26],
- The synthesis method (Corey-Seebach synthesis), on which the production of a commonly used germanium-based compound (Ivocerin^TM^) is based, is complicated and involves several stages, which affects the reaction efficiency. The challenge undertaken by Frűhwirt’s group is to develop a one-pot synthesis method [27],
- Tetraacylgermanates have poor solubility; the perception is to appropriate substituents [27],
- Germanium-based derivatives showed little or no toxicity. It was also demonstrated that there was no effect on the occurrence of gene mutations [24].

**Figure 3:**
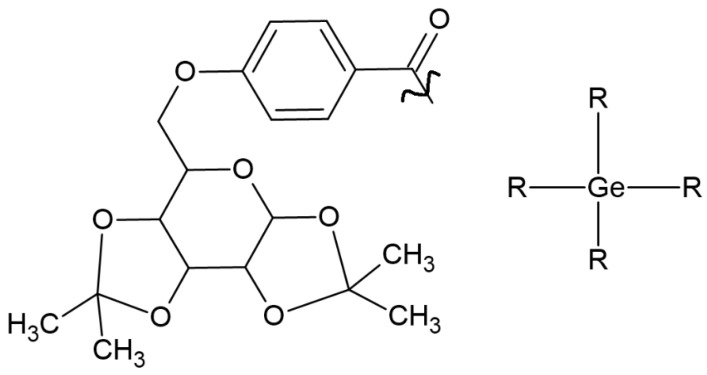
Chemical structure of tested compound based on germanium, with increased solubility [28].

Ivocerin^TM^ is a widely available photoinitiator based on bis(4-methoxybenzoyl) diethylgermane. Despite its many advantages, its weakness is low solubility. To expand the possibility of its implementation in medicine, Professor Haas’s group (1) developed a one-pot synthesis method, (2) maintained low toxicity, (3) extended the absorption band, and (4) improved solubility by introducing D-galactose. One compound (Figure 3) that possesses high solubility in water and an absorption spectrum between 350 and 450 nm was selected [28].

The future use of germanium-based compounds needs an increase in water solubility [29]. To the best of the author’s knowledge, the use of photoinitiators prepared based on germanium may be an alternative to commercially used photoinitiators. The various advantages they offer, the key one arguably being no toxicity for cells, are weighed by the high cost at which they arrive. Nevertheless, cost appears as a secondary feature in the medical realm, so their implementation here may be explained.

**Table 2:** Commonly used naturally origin photoinitiators.

Name of Substances	Chemical Structure	Type of Photoinitiator	Uses in Medicine	Additional Information	**Literature**
Riboflavin (7,8-dimethyl-10-ribityl-isoalloxazine)	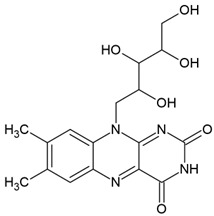	Type II	hydrogels in drug delivery, tissue crosslinking, dermal filler, cellular scaffolds	yellow pigment, water-soluble and heat-stable, can be found in milk, eggs, fish, vegetables (broccoli, asparagus), no toxic for humans, absorbs in range between 220 and 450 nm with maximum absorbance: 223 nm, 267 nm, 373 nm and 444 nm, used as a photoinitiator in the absence of DMAEMA (co-initiator) and with the use DPIC (accelerator)	[20,22,30,31]
Coumarins (2H-1-benzopyran-2-one)	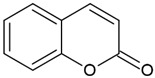	Type I and II	3D printing, drug delivery systems, tissue engineering, biomaterials, dental resin	extracted from bacteria (*Streptomycces*) or fungal (*Aspergillus*) and plants (green tea, cloudberry, tonka beans), absorption spectra in the range between 270 and 510 mm, maximum absorbance at 330 nm,	[32,33]
Flavones	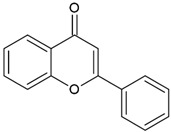	Type II	3D printing	found in vegetables (celery, parsley), fruits (citrus fruits), flowers (chamomile), greenish—yellow colour, absorption spectra in the range between 350 and 470 nm, maximum absorbance at 405 nm	[20,21,34]
Curcumin (E,E)-1,7-*bis*(4-hydroxy-3-methoxy-phenyl)-1,6-heptadiene-3,5-dione	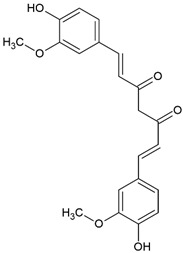	Type II	hydrogels, photodynamic therapy, antibacterial coatings	orange-yellow pigment, insoluble in water, is extracted from turmeric, absorb light in a range between 350 and 750 nm with maximum absorbance at 436 nm, is used as a photoinitiator in the absence of an electron donor—for example, DPI	[20,35,36,37,38]
Chalcones (1,3-diphenyl-2-propen-1-one)	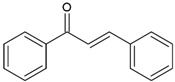	Type II	3-D printing dental materials	yellow, orange and brown colour, absorb light in the range between 220 and 270 nm and 340 and 390 nm with maximum absorption at 423 nm, 363 nm, 362 nm and 344 nm	[20,21,39,40]
Naphthoquinones (1,4-naphthoquinones)	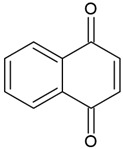	Type I and II	polymerization of acrylates	orange, yellow, red, purple colour, extracted from a tropical shrub Lawsonia inermis maximum absorption in visible light region—420 nm	[20,41,42,43]

**Table 3:** Commonly used synthetic origin photoinitiators.

Name of Substances	Chemical Structure	Type of Photoinitiator	Uses in Medicine	Additional Information	**Literature**
CQ (camphorquinone)	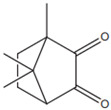	Type II	dental resin	curing by using a blue wavelength spectrum of light (from 430 to 510 nm) with maximum absorption peak of 468 nm (molar extinction coefficient—28 L/mol generating two radicals (amino and alkyl), rate of conversion of resin composites is between 35 and 77% Advantage: sensitive to light in the visible range Disadvantage: yellow colour—it negatively affects the appearance of dental resin, unreacted CQ has a toxic effect on pulp cells, a low value of molar extinction coefficient	[17,19,44,45,46,47,48]
TPO (2,4,5–trimethylbenzoyldiphenylphosphine oxide)	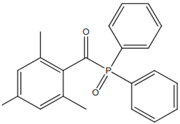	Type I	dental resin, hydrogels	the absorption spectrum is between 380 and 425 nm with maximum absorption peak at 380 nm, Advantage: no yellowing effect and higher colour stability, high molar absorption coefficient (520 L/mol) Disadvantage: it may be polymerized only as thick layers that increase the degree of shrinkage and use a UV light source	[44,47,49]
BAPO (*bis*acylphosphine oxide)	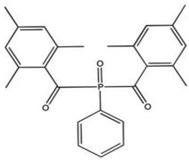	Type I	dental resin,	absorption spectrum between 365 and 416 nm and maximum absorption peak at 370 nm (molar extinction coefficient—870 L/mol, generate 4 types of radicals, Advantage: white colour which has a positive effect on dental resin, no need for the presence of co-initiator, Disadvantage: need to use a halogen lamp,	[44,50]
BP (benzophenone)	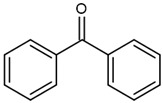	Type II	dental resin,	absorption spectrum between 320 and 370 nm and 240 and 300 nm and maximum absorption peek at 294 nm, Advantage: low cost and efficiency Disadvantage: need to use a light source with UV spectrum	[17]
PPD (1-phenyl-1,2-propanedione)	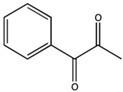	Type I and II	dental resin	absorption spectrum between 350 and 500 nm, and maximum absorption at 398 nm (molar extinction coefficient—150 L/mol, Advantage: positive effect of colour of dental resin,	[44]
IVO (Ivocerin^®^) *bis*-(4-methoxybenzoyl)diethyl-germane (Ge-3)	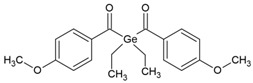	Type I	dental resin	Absorption spectrum between 390 and 445 nm, with maximum absorption at 418 nm Advantage: no cytotoxicity, White colour, High initiation rate Disadvantage: high cost, low solubility	[17,51,52,53]

## 3. Application of Photoinitiators Used in Medicine

### 3.1. Dentistry

A milestone for light-cured dental fillings was made in 1975. This resulted from the introduction of orthophosphate acid to the acrylate composition (1955) and bis-GMA resin as the primary ingredient of dental fillings. Currently, this field offers excellent development opportunities to improve resistance to chemical factors and biomechanical properties [54]. The main directions of studies are improving aesthetic parameters, reducing polymerization shrinkage and increasing the depth of hardening of dental composites.

The light-curing composition includes

**Figure 4:**
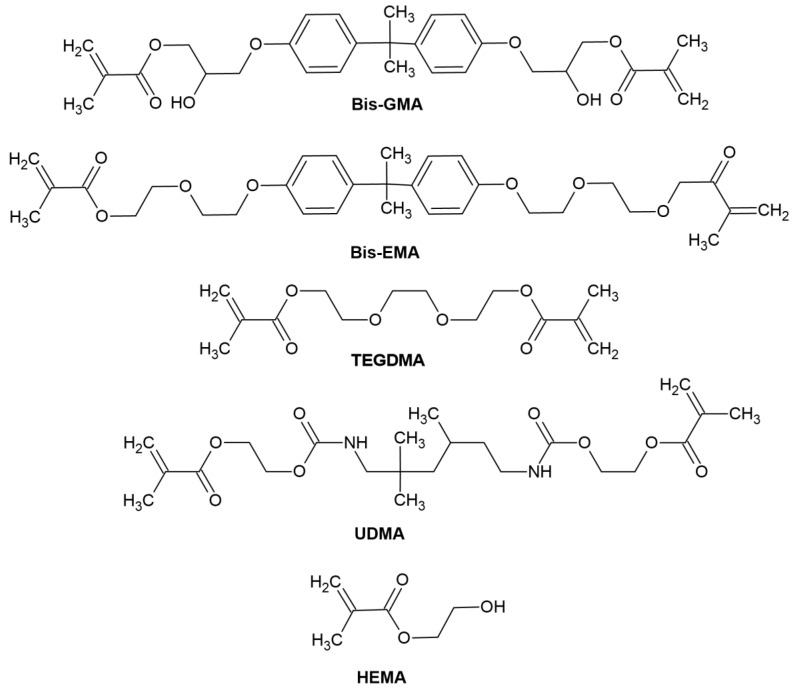
Reproduced by [54]. Chemical structure of monomers used in dental materials.

- Organic resin matrix: organic monomers—among which the most frequently used include bis-GMA ((2,2-bis [4-(2-hydroxy-3-methacryloyloxypropoxy)]-phenyl propane) or bisphenol A-glycidyl methacrylate), bis-EMA (ethoxylated bisphenol A glycol dimethacrylate), TEGDMA (triethylene glycol dimethacrylate), UDMA (urethane dimethacrylate), HEMA (hydroxyethyl methacrylate), photoinitiator or photoinitiator system and stabilizers to extend durability [54]. The chemical structure of monomers is shown in Figure 4.
- Inorganic fillers, which increase strength and abrasion resistance. It is most often glass or silica,
- A coupling agent, which combines all the mixture’s ingredients with the organic matrix [17].

Curing the mixture (transforming it from a liquid to a solid) can occur due to three processes: chemical, under the influence of light or a combination of the abovementioned processes. Later in the article, we will concentrate on the method using light. The prerequisite for the implementation of light is employing a photoinitiator.

When talking about photoinitiators, one ought to briefly outline the history of commonly used photoinitiators. In 1978, a camphorquinone (CQ), a type 2 photoinitiator, most often used with a co-initiator, such as a tertiary amine, was introduced at a broader scale. However, the yellow colour of CQ diminishes the aesthetic values of dental resin. Moreover, unreacted CQ may cause cytotoxic effects on pulp cells. Although the concentration pattern positively affects the mechanical properties and the degree of conversion, it reduces the aesthetic parameters. Additionally, the presence of amine as a co-initiator results in faster yellowing over time [17,44,45,46,47].

Wang’s team dealt with the impact of concentration on the physicochemical parameters of dental resin. The parameters considered in the publication are particularly pertinent when assessing photoinitiators: degree of conversion (DC), mechanical properties, and curing depth. The previously stated proposition was confirmed that an increase in the concentration of the photoinitiator has a significant impact on the DC and mechanical properties of dental resin (Bis-GMA/TEGDMA—70:30). The concentrations taken into account are 0.1; 0.2; 0.5; 1.0; 2.0 wt% CQ. The concentration chosen best reflected the assessed parameters—0.5 wt% CQ. Above this value, no significant impact on DC was observed, and even a deterioration of mechanical parameters was observed. This is confirmed by the results presented in the Table 4 [55].

**Figure 5:**
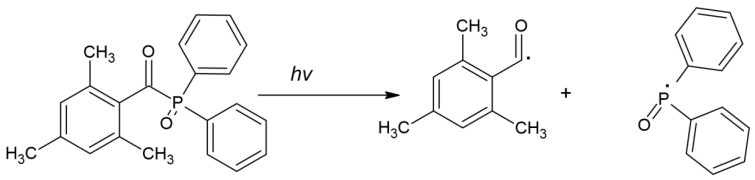
Chemical structure of cleavage of TPO [44].

Reducing the negative impact of CQ led to searching for new PIs. Among them, the following can be found:

(a) TPO can act as a one-component PI, but exploiting TPO in combination with CQ is also known, which shows a positive effect [47,49]. The cleavage of TPO is shown in Figure 5.

Scientists aim to improve the effectiveness of TPO by optimizing reaction conditions to find a “golden mean” in terms of effectiveness and obtaining the smallest number of adverse effects, combining TPO with another photoinitiator or modifying its chemical structure.

Kowalska et al. studied the effect of concentration of TPO on the typical parameters of dental fillings. The research results confirmed several vital hypotheses: (1) an increase in TPO concentration has a positive effect on the improvement of hardness; (2) the diametral tensile strength testing confirmed higher values for systems containing TPO (concentration above 0.5% by weight) than for the CQ/DMAMEA test systems; (3) evaluating the results of the three-point strength test indicate that there are no significant differences between specific compositions; (4) the photoinitiator concentration also influenced the colour of the composites. It was found that the higher the concentration, the closer the colour was to yellow. To sum up, the concentration that best presents the values of the assessed parameters is 0.75 wt. However, further trials, including cytotoxicity, are necessary to use these tests in industrial production. [56].

**Figure 6:**
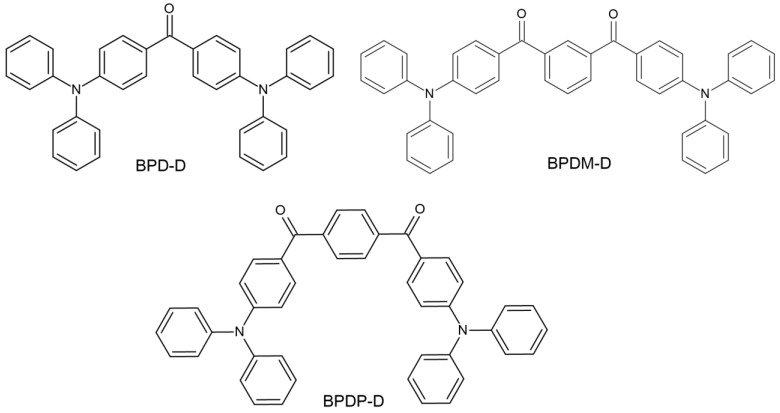
Chemical structure of new compounds [57].

(b) BAPO—The advantages of this PI reveal a degree of conversion and polymerization rate similar to that of the CQ/amine system. Additionally, the presence of a co-initiator is redundant [36]. Experimental work by Lim et al. [50] confirmed the abovementioned content. However, they indicate the need to conduct additional studies on this photoinitiator, examining the interaction between photoinitiators and monomers in the composites.
(c) BP—A shift in the absorption spectrum towards longer wavelengths was started by Huang’s team. Three new compounds were synthesized based on the BP skeleton. Its chemical structure is presented in Figure 6. The experiment obtained compounds with good solubility in organic solvents and the TMPTMA (trimethylolpropane trimethacrylate) monomer. The modification of the chemical structures of benzophenone resulted in a shift of the absorption spectrum to approximately 380 nm and an increase in the molar extinction coefficient. The effectiveness of new photoinitiators in combination with the abovementioned monomer in the photopolymerization process was also demonstrated. This opens up new possibilities for BP modification [57].

(d) PPD—Connecting with CQ positively reduces the degree of yellowing. Its presence also improves the physicochemical properties of the obtained polymer [44,58]. Dressano’s team studied three-component systems (CQ, PPD and DPI salts—diphenyliodonium hexafluorophosphate) and their effect on the physical and chemical properties of the dental resins. PPD is supposed to reduce the yellowing effect, and DPI is supposed to improve the reaction efficiency by regenerating inactive CQ molecules and replacing them with active phenyl radicals. The best concentration of CQ-PPD is 0.5 mol% and DPI is 1 mol%. This system showed low water absorption, low solubility and good mechanical properties—these features are desirable in dental resin formulations [58].

Several papers regarding dental composites in which CQ was set as a reference point have been published by Pyszka’s group. The tested initiators were based on the quinoxaline skeleton, and the co-initiators were acetic acid derivatives. This treatment was intended to eliminate the presence of amines, which have a toxic effect. The features assessed during the experiment are as follows: (1) Exothermic photopolymerization—which is extremely important in terms of pulp damage. The four newly formed compounds were subjected to the photopolymerization process and it was found that a hard polymer could be obtained after just 30 s. It has been proven that none of the new photoinitiator systems employed exceeds the threshold of the average temperature increase responsible for the occurrence of pulp inflammation −5.5 °C. For comparison, compositions with CQ exceed the mentioned threshold but are lower than the threshold of 11.2 °C, which is responsible for 60% of the pulp surface undergoing irreversible changes. (2) Surface morphology observed using a confocal optical microscope. Combined with commercially available dental fillers (DF1, DF2, DF3), they showed surface uniformity. (3) Compressive strength, which is significant in terms of testing the resistance of the material when chewing food. Regardless of the type of photoinitiator used (Ph1 (dibenzo[a,c]phenazine), Ph2 (benzo[a]phenazine), Ph3 (11H-indeno [1,2-b]quinoxalin-11-one), or Ph4 (6H-indolo [2,3-b]quinoxaline)), better strength was obtained than the compositions composed of CQ and DF1 [59].

**Figure 7:**
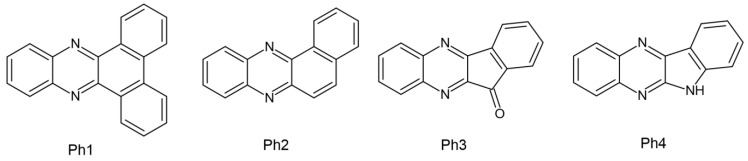
Chemical structure of photoinitiators based on quinoxaline skeleton [30]. (Ph1) dibenzo[a,c]phenazine, (Ph2) benzo[a]phenazine, (Ph3) 11H-indeno [1,2-b]quinoxalin-11-one, (Ph4) 6H-indolo[2,3-b]quinoxaline.

The chemical structure of the abovementioned compounds is shown in Figure 7.

It was established that the proposed photoinitiators could be used in dental materials. However, these compounds require additional studies—primarily toxicity assessment [59].

**Figure 8:**
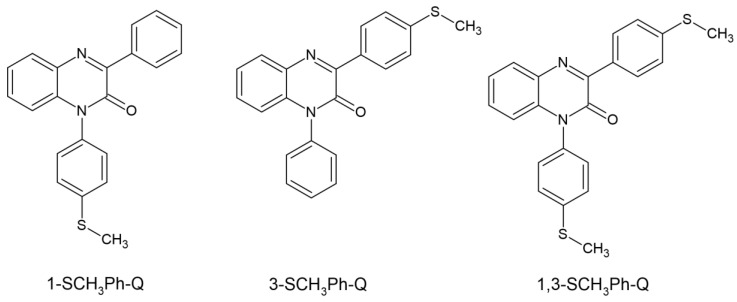
Chemical structure of new tested photoinitiators by Ortyl’s group [60].

Ortyl’s team dealt with the matter of quinoxaline and its two derivatives. The three newly synthesized compounds showed absorption in the range of up to 430 nm, with an absorption maximum from 363 to 371 nm. The electrochemical and thermodynamic properties were examined. Quinoxaline-2 derivatives were classified as type II photoinitiators and electron donors acting with iodonium salt as electron acceptors. The kinetics of photopolymerization was studied via the FT-IR method. The selected system chosen was 3-SCH3Ph-Q/IOD, it demonstrated the best properties when used in dental fillings. Its convenience was assessed by examining the photopolymerization process of the UDMA/TEGDMA mixture. The selected system was characterized by low shrinkage—2.8% and a hardness of 82 +/− 4. The chemical structure of studied compounds is shown in Figure 8 [60].

Another direction in the study of dental materials is focused on the germanium derivatives. The year 2008, when Liska’s team patented these compounds as initiators, can be considered the beginning of work on initiators containing germanium in their structure [61]. Two new benzoyl germanium derivatives exhibited an absorption maxima in the visible range, low toxicity, and good solubility in monomers, no yellowing effect were proposed. Their disadvantages include poor solubility in water and inability to use in cationic polymerization. One of the compounds—DBDEGe—showed particularly advantageous features: short curing time and considerable depth of hardening [24,62].

**Figure 9:**
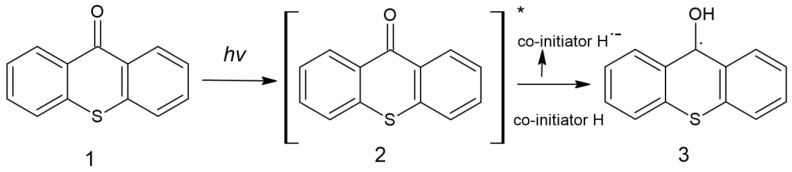
Formation of radicals by thioxanthone [63].

Due to its aromatic structure, thioxanthone is used in photoactive processes; the study on using TX as a photoinitiator was initiated in 1981 by Amirzadeh and Schnabel. Since then, it has been used in the production of coatings [63]. It is possible to find information about thioxanthone-based compounds and their use as type II photoinitiators, most often combined with amines and use both in free and cationic photopolymerization [8]. The formation of free radicals initiating the polymerization process is presented in Figure 9 [63].

**Figure 10:**
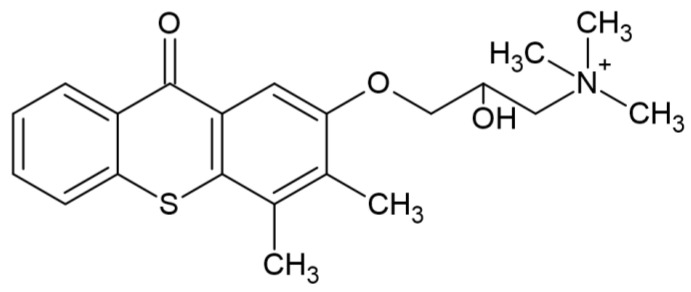
Chemical structure of compound based on thioxanthone skeleton [64].

After absorption of the quantum of light, the TX (1) molecule is excited, (2) and then reacts with the proton donor (usually an amine), generating a radical (3) as a result of decomposition. An example of a photoinitiator that can be used in the production of dental fillings is (2-hydroxy-3-(3,4)-dimethyl-9-oxo-9H-thioxanthen-2-yloxy)-N,N,N-trimethyl-1-propanium chloride (QXT) tested by Ely et al. with the chemical structure shown in Figure 10 [64]:

Due to its absorption in the UV range, it was combined with CQ and various co-initiators. Adding QTX in combination with iodonium salt or organic acid derivatives can improve the degree of polymerization of CQ-based resins [64].

**Figure 11:**
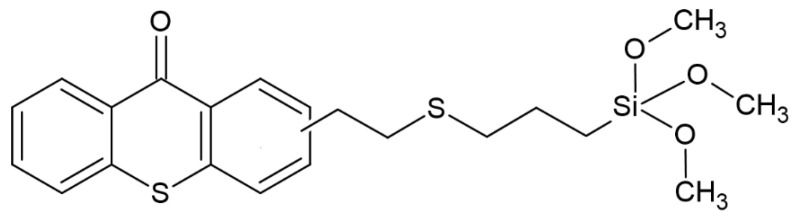
Chemical structure of obtained compound based on thioxanthone skeleton [65].

Recently, another group of scientists has shown interest in thioxanthones (TX); the experiment aimed to synthesize TX derivatives with a siloxane molecule. The effectiveness of the resulting system was tested in combination with iodonium salt, obtaining an effective photoinitiator operating at wavelengths of 385, 405 and 455 nm. The proposed system is effective in both radical and cationic polymerization. The structure of the studied compound is shown in Figure 11. An additional effect achieved is the effect of low diffusion—up to 2%. For example, further use of this compound is indicated for dental purposes [65].

### 3.2. Hydrogels

The year 1950 can be considered the opening of work on hydrogels; at that time, Katchalsky’s team synthesized hydrogels for the first time [66]. Their relevance is constantly increasing, which can be confirmed also by the number of publications reviewing the matter that have appeared. Even without going into details, by entering the phrase “hydrogels” in the PubMed database, one can notice a significant increase in interest in this area. In 2000, 293 publications were found; as of mid-2024, the number was 5188.

In short, hydrogels are polymeric materials with a three-dimensional structure that can absorb large amounts of water. The water content can be up to 95% [67]. Their prospect of biomedical use results from their characteristic features: ease of use, biocompatibility, low toxicity and biodegradability [68].

The main requirements for photoinitiators if they will be used in hydrogels are no toxic effect on cells, solubility in water, compatibility with a light source, and thermal stability. For biomedical applications, radical rather than cationic photoinitiators are preferred [69].

For two seemingly ideal photoinitiators, TPO-Li and Irgacure 651, following reviewing them against general requirements, it was necessary to limit their implementation due to the European Chemical Agency’s findings on TPO toxicity [70]. Further, Irgacure demonstrates poor water solubility; these findings indicate the need for launching intensive studies in this field.

**Figure 12:**
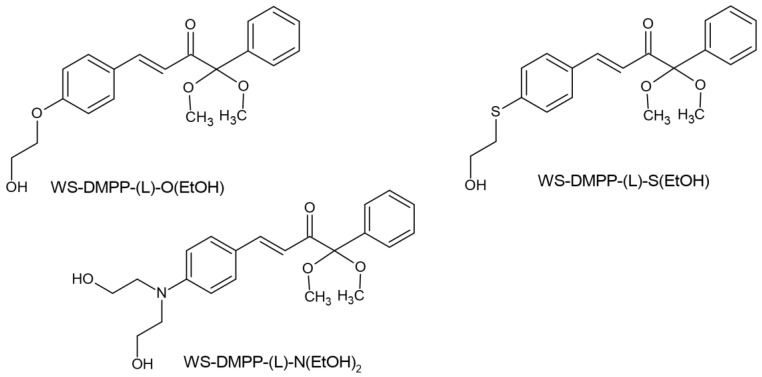
Chemical structure of compound tested by Tomal et al. [69].

Significant progress in the search for new photoinitiators can be seen in the recently published papers by Tomal’s team. The team’s work included synthesizing five new, unknown benzoin-ketal-based derivatives and determining their spectroscopic properties, photo-fragmentation mechanism, cytotoxicity and water solubility. Among this group of photoinitiators, one, marked WS-DMPP-(L)-N(EtOH)_2_, ((E)-1,1-dimethoxy-1-phenyl-4-[4-[bis(2-hydroxyethyl)amino]phenyl]-but-3-en-2-on), showed the most promising results; it absorbs up 500 nm and shows maximum absorption at 408 nm and is characterized by a high molar extinction coefficient of 30,311 L/mol. The introduction of the (2-hydroxyethyl)-amine (-N(EtOH)_2_) group increased the solubility from 0.14 g/L (Irgacure 651) to 3.32 g/L. This photoinitiator showed photoactivity even at a concentration of 0.1% by weight. It has revealed three photoinitiators that offer the possibility of further use. The chemical structure of tested compounds is shown in Figure 12 [69].

Chen’s team began modifying the carbazole skeleton to obtain 3D antibacterial hydrogels. Their biological activities justify their medical use: antimicrobial, antiepileptic or antioxidant. The study aimed to improve the solubility of carbazoles by introducing the 1,4-butanesultone and nitro groups. Solubility levels of 7% and 9% were achieved. The absorption bands range from 200 to about 560 nm. Further, a photoinitiation system consisting of carbazole/IOD/TEA offered a monomer conversion of 98% [71].

**Figure 13:**
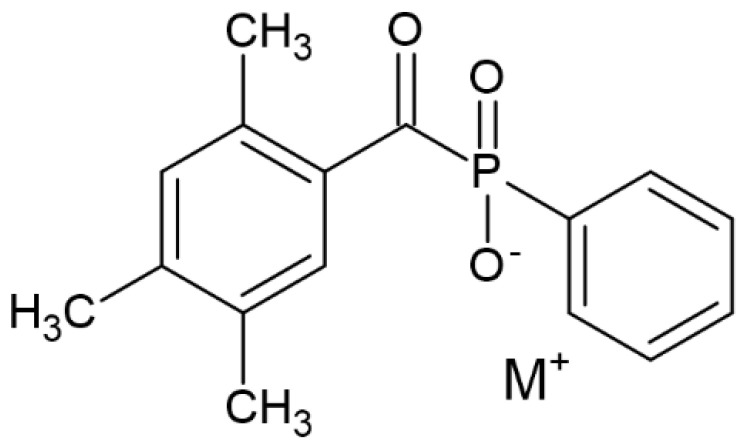
Chemical structure of tested compound by Wang [72].

To balance the poor solubility and low photoreactivity in the 385–405 nm range of commonly available photoinitiators (Irgacure 2959), Wang and co-workers developed a method to improve water solubility. The simple technique developed for that purpose intended to produce a metal-phenyl(2,4,6-trimethylbenzoyl)phosphinate complex (M-TMPP) as a photoinitiator with excellent solubility and biocompatibility. Lithium, magnesium and sodium were tested as metals. In addition to the abovementioned features, which are considered advantages, the process turned out to be very easy to replicate. It assumes the occurrence of only two stages—TMPP synthesis and salinization—to introduce the appropriate metal. The structure of the obtained compound is presented in Figure 13 [72].

The obtained compounds were characterized by high solubility (15.5 g/L—Mg—TMPP, 40.5 g/L—Li—TMPP, 49.5 g/L—Na—TMPP). They absorb in the range 385–405 nm and generate radicals that make them useful in 3D printing (DLP). Toxicity tests also revealed the lack of toxic effect for values in the range of 1–40 mmol/L. Finally, the usefulness of photoinitiators in 3D printing was confirmed using acrylamide-monomer as a hydrogel precursor. The results confirmed the possibility of using the obtained photoinitiators in biomedical applications [72].

### 3.3. Drug Delivery

One possible way to use hydrogels is drug delivery. The advantage of this system is the ability to control the release over time and place which results in fewer side effects. Other characteristic features of hydrogels include neutral pH, lack of scent and colour, non-toxicity, and no affinity for drugs, hence their high degree of release, and finally, the safety of the drug for the environment [73].

Gencoglu et al. provided an opportunity to prepare an effective photoinitiator that can be used in drug delivery. They used TX as the framework for PI, explaining it by its non-toxicity, water solubility and absorption spectrum in the UV-Vis range. The compound marked as p(PEGDA 575-TX) was characterized by good solubility in water and photochemical activity in the visible light range. The maximum absorption of the new photoinitiator is at 254, 270 and 404 nm in water [74].

### 3.4. Photodynamic Therapy

The scope of applications of photoinitiators is broad. Still, one that deserves highlighting is photodynamic therapy (PDT). Due to its minimal invasiveness, the technique has been used in the treatment of many types of cancer: skin, head and bladder. The cytotoxic effect on diseased cells results from the influence of light, oxygen and photosensitizer [35]. The requirements for photosensitizers are deep cell penetration determined by a high excitation coefficient of 600–800 nm, generation of reactive oxygen species, minimal toxicity to cells, and accumulation in diseased tissue. Curcumins seem to be a noteworthy group of compounds in polymer chemistry. Their wide use is due to their exceptional biological properties (antimicrobial, antifungal, antibacterial) and photoreactivity. Restrictions on the broader use of curcumins are limited by their low solubility in water [20]. Ding and others conducted a study on changing this property [75]. The compound obtained when attempting to combine curcumin (because of the esterification reaction) with carboxymethyl chitosan was characterized by good stability, solubility in water (9.67 mg/L) and continued suitability for antibacterial photodynamic therapy. Under the influence of 450 nm radiation, the resulting compound generated reactive oxygen species that could destroy bacteria.

### 3.5. Individualized Tablets

The riboflavin as a photoinitiator has been used in pharmacy to produce small quantities of tablets individualized in terms of drug content, possibly customizing therapy to a particular patient. The publication’s authors emphasize the possibility of specific use in pediatric and geriatric medicine, where adjusting the dose to weight/age is particularly important. The work aimed to compose an appropriate system for photopolymerization using the DPP (Daylight Polymer Printing) technique. Various amounts of water, PEG 400, PEGDA MW 575 and the photoinitiator—riboflavin—were tested. To obtain the optimal results, the influence of co-initiators, ascorbic acid and triethylamine on the final product’s final characteristics was determined. Riboflavin combined with triethylamine efficiently initiated the photopolymerization process, creating a system that released the drug well [76].

### 3.6. 3D Printing

The term “3D printing” often appears among the possibilities of using natural photoinitiators. Its presence can be seen in many industries. Therefore, it is worth mentioning here what characterizes this type of printing. Its implementation can be explained by many advantages: excellent resolution, high efficiency and appropriate surface finish [54]. Natural photoinitiators in 3D printing expand its value. Many of those compounds can be found in the food or medical industry as tissue implants. Due to their multiplicity, one can also design their use in the entire range of UV-Vis light.

Among the publications on this topic, one can find research on the usefulness of flavones. The studies by Mousawi [77] provided vital information. The effectiveness of five flavone derivatives (flavone, 6-hydroxyflavone, 7-hydroxyflavone, chrysin and myricetin) was tested in the presence of co-initiators such as amine derivatives and iodonium salt. Hydroxyflavones showed a high ability to initiate polymerization. Their effectiveness in 3D printing was determined.

## 4. Conclusions

Regardless of the type of photoinitiator presented, its structure or current application in the biomedical field, the most important are the features it presents. These include biocompatibility, lack of cell toxicity, simple synthesis, high efficiency, and light absorption in the visible range and solubility in water. In view of the above, a group of photoinitiators of natural origin may have great potential for the future and can be particularly useful. This group has great potential expressed by many substances found in natural sources. Their modifications may be an opportunity to develop novel and safe initiators for application in the medical field. Further research related to the dependence of the structure on the absorption spectra will, in the future, allow for better design of photoinitiation systems tailored to a given application.

## Figures and Tables

**Table 1:** Comparison of polymerization methods. √—advantage, ×—disadvantage, and ±—intermediate. Based on [7].

Parameter	Thermal Polymerization	Photopolymerization
Chemical resistance	×	√
Variety in formulation	√	±
No substrate damage	±	√
Low curing temperature	×	±
Operational cost	×	√
Formulation cost	√	×
Capital cost	×	√
Cure rate	×	√
Skill level required	√	±
Non-solvent-releasing	√	±
Energy consumption	×	√
Radiation hazard	×	√
Fire hazard	×	√

**Table 4:** CQ concentration vs. maximum DC and DC rate [55].

CQ Concentration (wt.%)	Max. DC (%)	Max. DC Rate (%s^−1^)
0.1	54.177	5.081
0.2	62.912	10.464
0.5	69.577	15.753
1.0	71.825	16.258
2.0	75.450	12.643
